# The effectiveness of moxibustion for stable angina pectoris: A meta-analysis and trial sequential analysis protocol

**DOI:** 10.1097/MD.0000000000016829

**Published:** 2019-08-16

**Authors:** Yili Zhang, Yuan Li, Juan Wang, Nannan Tan, Junjie Liu, Kangjia Du, Miao Zhang, Yong Wang, Huihui Zhao, Wei Wang

**Affiliations:** a School of Traditional Chinese Medicine; b Dongzhimen Hospital, Beijing University of Chinese Medicine; c Xiyuan Hospital, China Academy of Chinese Medical Sciences; d School of Life Science, Beijing University of Chinese Medicine, Beijing, China.

**Keywords:** cardiovascular diseases, Chinese medicine, evidence-based medicine, non-drug therapy

## Abstract

**Backgrouds:**

Moxibusion is a famous traditional Chinese medicine (TCM) treatment, which can be used to treat stable angina pectoris for many years. We will conduct this study to explore the efficacy and safety of moxibustion as an additional therapy and to provide more reliable evidence for clinical practice.

**Methods:**

We will go through 8 databases until July 2019 to identify related randomized controlled trials that compared moxibustion with the control group. The main result is the clinical effective rate. RevMan (V.5.3) and test sequential analysis (V.0.9) will be used for mata analysis and trial sequential analysis.

**Results:**

This study will provide a high-quality synthesis of current evidence of moxibustion and we have a specific opportunity to determine the efficacy and safety of moxibustion in patients with stable angina pectoris.

**Conclusions:**

This study will explore whether or not moxibustion can be used as one of the non-drug therapies to prevent or treat stable angina pectoris, especially in the elderly population with related risk factors.

**Registration number:**

CRD42018112830

## 1 Introduction

Cardiovascular disease (CVD) is the most important factor in the overall mortality rate in the general population. Among the risk factors of elderly patients with CVD, aging is one of the main characteristics different from that of non-elderly patients.^[1,2,3]^ More than half of all patients who die of CVD are over the age of 70.^[4]^ Stable angina pectoris (SAP), the most prevalent symptom of CVD,^[5]^ is a clinical syndrome that greatly compromises a patient's life quality and longevity, usually caused by fatigue or stress and relieved by rest or nitroglycerin.^[6]^ With the gradual development of China's aging society, the aging process is accelerating.^[7]^ The prevalence of stable angina pectoris in mainland China is about 3.6%.^[8]^ At the same time, previous studies have shown that the incidence of angina pectoris in the United States is 12.3/1000 person-years, and the prevalence rate is 20% among men over 60 years old. Also, a similar situation is in Europe.^[9,10]^ The annual mortality rate of SAP patients was 1.2 ± 2.4%.^[11]^ This syndrome places a heavy burden on the health-care system, so its management is a priority and requires further research and noble treatment.

The current guidelines suggested that drugs can be divided into first-line drugs (β-blockers, calcium channel blockers, etc) or second-line drugs (long-acting nitrate, evabradine, nicorandil, etc). Although there are a variety of treatment options for patients, most patients will still expose to the side effects of the drug,^[12]^ such as headache,^[13]^ mental fatigue^[14]^ and harm with myopathy and hepatotoxicity.^[15]^ Non-drug therapy as a secondary option is usually chosen by the patient. Moxibustion is one of the main components of therapies in traditional Chinese medicine (TCM). The heat produced by burning moxibustion wool stimulates acupoints,^[16,17]^ which has been widely adopted in treating angina pectoris in clinical practice^[18]^ and generally accepted and recognized worldwide because of the clinical effectiveness.^[16]^ Previous systematic reviews and randomized controlled trials (RCTs) have shown that moxibustion has potential benefits for certain cardiovascular diseases, including hypertension,^[19]^ stroke rehabilitation,^[20]^ and so on. Moreover, moxibustion has been reported to prevent inflammation, organ dysfunction^[21,22]^ and hormonal imbalances.^[23]^ It activates specific receptors, heat-sensitive immune cells, and heat shock proteins, and produces a variety of physical effects by activating warming and dredging functions. Hyperthermia through neural and humoral pathways and subsequent behavior can induce further effects on specific target organs and body systems.^[24]^

However, from the point of view of evidence-based medicine, the efficacy and safety of moxibustion on SAP are still controversial. Several RCTs have been performed to evaluate the clinical benefits of moxibustion in the treatment of SAP. Although the number of clinical trials related to the effectiveness and safety of moxibustion soars, there is no systematic review and meta-analysis of moxibustion on SAP. Therefore, we have a specific opportunity to conduct this meta-analysis to determine the efficacy and safety of moxibustion in patients with SAP. In addition, we examine whether the current evidence is robust and conclusive by using trial sequential analysis (TSA).

## 2 Material and methods

This study has been registered at PROSPERO (registration number: CRD42018112830; http://www.crd.york.ac.uk/PROSPERO). This meta-analysis will be based on the preferred reporting items for the systematic review and meta-analysis of the (PRISMA) project.^[25]^

### 2.1 Inclusion criteria

#### 2.1.1 Type of studies

We will include all RCTs to explore the specific efficacy and safety of moxibustion in the treatment of SAP. Cross-trials, quasi-RCT, animal experiments and other studies that were repeatedly published or did not have access to complete data will be excluded.

#### 2.1.2 Types of participants

Participants who meet the diagnostic criteria of SAP or stable coronary heart disease will be included.^[26,27,28,29]^ All included participants in this review regardless of their age, race, and gender.

#### 2.1.3 Types of interventions

We will only include studies which interventions involved a flaming moxa wool at acupuncture points, pain points or trigger points, and were described as moxibustion. Conventional therapies with moxibusiton that was addictive to the active treatment will be also included. However, studies that compare the efficacy of different forms of moxibustion will be excluded as this was differing from the focus of the review.

#### 2.1.4 Types of comparisons

The control groups which can verify the effectiveness of the moxibustion as a monotherapy or in combination with conventional therapies will be considered. For instance: moxibustion versus anti-SAP drug, (moxibustion plus anti-SAP drug) versus (anti-SAP drug), moxibustion versus no treatment.

#### 2.1.5 Types of outcomes

The primary outcome is the clinical effective rate, which could be categorized as mainly 4 parts including cure, markedly effective, effective, or invalid according to the Guideline for Clinical Trials of New Patent Chinese medicines (GCTNPCM).^[30]^ The evaluation standards were based on clinical symptoms and electrocardiogram (ECG) changes (mainly ST-segment depression). The secondary outcomes mainly including nitroglycerin use (the stopping or reducing rate of the nitroglycerin), ECG changes (the effective rate of ECG), symptoms of angina pectoris, serum lipid, hemorheology, and adverse effect.^[31,32]^

### 2.2 Search strategy

We will go through the following 8 databases from inception to July 2019: Cochrane Library, Medline (via PubMed), Web of Science, Clinicaltrials.gov, Chinese National Knowledge Infrastructure (CNKI), Wanfang Data, Chinese Biomedical Literature (CBM), the VIP Chinese Scientific Journal Database (CQVIP). “Moxibustion” will be combined with “acupoint”, “traditional Chinese medicine”, “coronary heart disease”, “randomized controlled trial”, “SAP”, and “angina pectoris” respectively for literature search. The search strategy for selecting the fields of topic, title or abstract was different referring to the characteristics of databases. The full list of the search strategy for PubMed as follows:

#1. (((moxibustion [Title/Abstract]) OR moxa [Title/Abstract]) OR acupoint [Title/Abstract]) OR traditional Chinese medicine [Title/Abstract].
#2. (((((((cardiovascular[MeSH Terms]) OR cardiovascular[Title/Abstract]) OR cardiac[MeSH Terms]) OR cardiac[Title/Abstract]) OR angina pectoris [MeSH Terms]) OR angina pectoris [Title/Abstract]) OR coronary heart disease [Title/Abstract].
#3. (((random [Text Word] OR randomized [Text Word]) OR control [Text Word]) OR controlled [Text Word]) OR trial [Text Word]
#4. #1AND#2AND#3 Filters: Clinical Trial; Humans.
Modified search strategy will be used for other electronic databases.

### 2.3 Selection of studies

**Figure 1:**
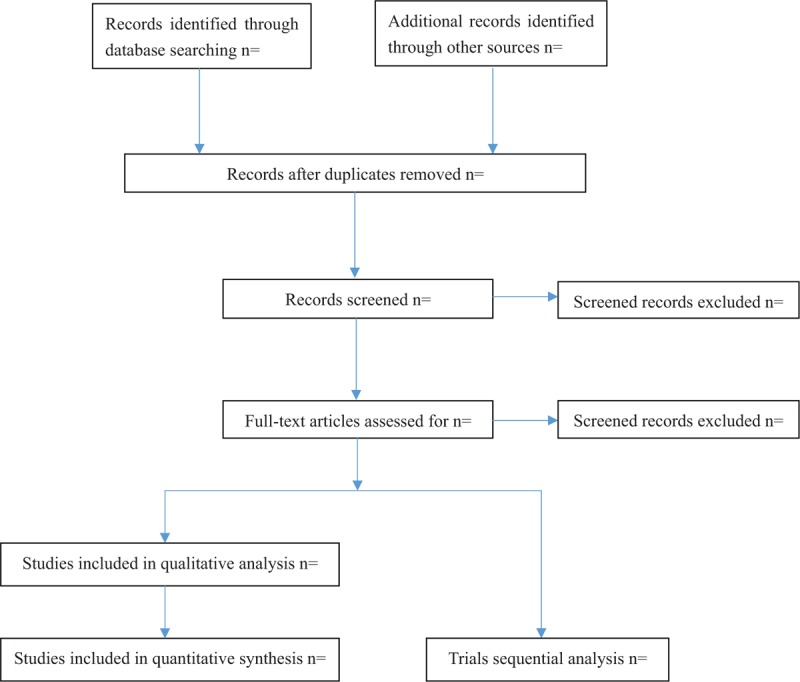
Flow diagram of studies identified.

Search results are imported from the original database into NoteExpress V.3.2.0. Two evaluators (ZYL and LY) will independently evaluate the eligibility of retrieval studies on the basis of inclusion criteria. Titles and abstracts will be reviewed to exclude manifestly inappropriate publications for preliminary research options, and reading the full version is the next step in further assessing the inclusion of the study. The 2 evaluators then will cross-check the selection results. Any differences will be resolved by consensus. Further arguments will be arbitrated by a third commentator (ZHH and WW). The details of the selection process are shown in Figure 1.

### 2.4 Data extraction

Two reviewers (ZYL and LY) will use predefined data collection forms to extract data independently. The table consists of 4 main areas:

(1) general information (for example, title, author, reference, year of publication);
(2) intervention and comparison (dose, route, and time);
(3) patients (for example, baseline characteristics and diagnostic criteria);
(4) results (e.g., ECG, hemorheology, nitroglycerin withdrawal rate).

In the event of disagreement, arbitration will be conducted through discussion or through the third reviewer (ZHH and WW).

### 2.5 Assessment of risk of bias

Each of the included trials will be evaluated and reported the risk of bias by using the Cochrane Collaboration's tool for assessing risk of bias.^[33]^ Two reviewers will input these included studies into RevMan software (V.5.3)^[34]^ and assessed the methodological quality. Trials rated as high risk of bias in 1 or more areas will be defined as “high risk”, while low bias risk in all areas will be rated as “low risk”. Otherwise, the trials will be seen as “the risk is not clear”. The 2 evaluators independently will assess the quality of the methodology and resolve their differences through discussions by a third reviewer (ZHH and WW).

### 2.6 Data analysis

The Revman 5.3 software provided by the Cochrane collaboration Network will be used for data analysis. The random effect model will be used to determine the measured values of all merged results. For binary results, a merged RR with 95% CI will be used as a measure of the effect. For a continuous result with the same unit, the weighted mean difference (WMD), will be used when the result units are consistent. Potential heterogeneity across the included studies will be assessed by χ2 test and I^2^ test. If I^2^ ≥50% and *P* <.1, it suggests that there is statistical heterogeneity. If the number of included studies is less than 2 or heterogeneity is apparent, the results of our systematic review will be narratively reported. If the included studies with multiple arms, we will identify the relevant intervention and control groups, then combine the relevant groups into a single group before synthesizing the data.

### 2.7 Sensitivity analysis and subgroup analysis

If there is significant heterogeneity, the group will be divided into subgroups with similar characteristics according to the characteristics of the study, including sample size, treatment duration, and other relevant parameters, in order to explore potential sources of heterogeneity. If heterogeneity cannot be resolved (when the I^2^ statistic exceeds 50%) no meta-analysis will be performed.

### 2.8 Trial sequential analysis

TSA will be conducted to obtain the primary result. Cumulative meta-analysis might result in false-positive results (type I error) because of an increased risk of random error from sparse data and repeat significance testing.^[35]^ TSA could control the *P* value and widen the confidence intervals.^[36]^ Estimation of the required information size and trial sequential monitoring boundaries were the concepts and rationale combined by TSA. If the cumulative Z curve entered the futility area or crossed the trial sequential monitoring boundary, the anticipated intervention effect might reach a sufficient level of evidence, and further trials would not be necessary.

We calculated the required information size based on a relative risk reduction of −20% in clinical effective rate. The type I error (α) and power (1–β) were set as 0.05 and 0.80, respectively. The control event rates are calculated from the non-subglottic secretion suctioning group. The TSA will be conducted via TSA version 0.9 beta software (http://www.ctu.dk/tsa).

### 2.9 Ethics and dissemination

As individuals will not be involved, the ethical approval will not be required. The results of the study will be published in peer-reviewed journals and published at relevant conferences.

## 3 Discussion

Previous experiments^[37]^ have shown that moxibustion can protect myocardial cells from ischemia/reperfusion injury by activating the expression of endogenous myocardial protective substances and increasing the expression of cardiac gap junction protein. Moxibustion is also recommended as 1 of the treatment methods of SAP in clinical practice.^[38]^ Therefore, it is worth promoting moxibustion in the clinical prevention and treatment of myocardial ischemia with its simplicity, noninvasive, and acceptability.

The meta-analysis and trial sequential analysis might be the first time to show that moxibustion therapy whether or not has potential benefits for the treatment of SAP. However, before we accept it as an evidence-based treatment option in our clinical practice, we need more information to determine the benefit-harm situation of moxibustion on SAP. Furthermore, the quality of data must improve greatly if non-drug therapies are to assume a respected place in the contemporary health care.

## Author contributions

Visualization and software: Yuan Li, Kangjia Du.

**Data curation:** Yili Zhang, Yuan Li, Juan Wang.

**Formal analysis:** Yili Zhang, Yuan Li, Nannan Tan.

**Methodology:** Yili Zhang, Junjie Liu, Miao Zhang.

**Project administration:** Yong Wang, Huihui Zhao, Wei Wang.

**Software:** Yuan Li, Kangjia Du.

**Supervision:** Yong Wang, Huihui Zhao, Wei Wang.

**Validation:** Huihui Zhao, Wei Wang.

**Visualization:** Yuan Li, Kangjia Du.

**Writing – original draft:** Yili Zhang, Yuan Li.

**Writing – review & editing:** Juan Wang.

Yili Zhang orcid: 0000-0002-0044-8759.
